# Effect of Heat Treatment Process and Optimization of Its Parameters on Mechanical Properties and Microstructure of the AlSi11(Fe) Alloy

**DOI:** 10.3390/ma14092391

**Published:** 2021-05-04

**Authors:** Aleksandra Jarco, Jacek Pezda

**Affiliations:** Faculty of Mechanical Engineering and Computer Science, University of Bielsko-Biala, 43-309 Bielsko-Biała, Poland; jpezda@ath.bielsko.pl

**Keywords:** heat treatment, mechanical properties, Al-Si alloy, solution treatment, artificial ageing

## Abstract

The paper presents the results of study concerning the evaluation of the precipitation hardening parameters (temperatures and times of solution treatment and artificial ageing processes) having an effect on mechanical properties, and the change in the microstructure of the EN AC-AlSi11(Fe) alloy. Based on the obtained results and performed statistical analysis, regression equations and the response surface model in the form of spatial and contour plots were determined to illustrate the effects of solution treatment and artificial ageing parameters on the mechanical properties of the investigated alloy. The performed heat treatment had a positive effect on improving the mechanical properties of the alloy versus the initial state. The maximum increase in tensile strength was by 52%, in unit elongation by 56%, in Brinell hardness by 44% and impact strength by 88%. Furthermore, a favorable change was observed in the microstructure of the investigated alloy, especially regarding eutectic silicon precipitations, which underwent partial spheroidization and coagulation after the heat treatment.

## 1. Introduction

The Al-Si casting alloys (silumins) with the near eutectic chemical composition are characterised by very good castability [1], low casting shrinkage and susceptibility to form concentrated shrinkage cavity [2], as well as good mechanical properties. Furthermore, they are not prone to hot cracking, which allows to produce thin-walled castings with intricate shapes [3].

Iron, which has an adverse effect on alloy plasticity, tensile strength and castability due to the formation of brittle intermetallic phases, is the most common impurity present in Al-Si alloys [4,5,6]. Depending on alloy temperature and chemical composition, iron can, upon reaching its critical level, form intermetallic phases of different morphology. The most common of these are α(AlFeSi) phase polyhedral crystals in the shape of the so-called Chinese script and β(AlFeSi) phase platelets in the shape of needles [7,8,9,10]. The critical Fe content in alloy which triggers the formation of the detrimental β(AlFeSi) phase is approx. 0.75% Fe for 11% Si [11]. However, alloys used for high-pressure die casting can contain Fe levels even up to 1.5%, as the increased Fe content in alloy helps to minimize the costs of die-casting mould repair resulting from their use (mould cavity surface wear) [4].

Due to their very good casting parameters, high creep resistance and resistance to corrosion and abrasion, silumins are very popular materials, widely used in various industries. First of all, in the automotive sector, to manufacture heavy duty combustion engine pistons [12], transmission housings [13] and clutch housings, cylinder heads and engine blocks [14], and in the shipbuilding industry, as engine and fitting component castings, especially for components with required high leak-tightness [15,16].

The disadvantage of near-eutectic silumins is an irregular acicular or platelet-shaped form of eutectic silicon crystal precipitations, occurring especially during slow cooling down from the casting temperature. It conduces to the propagation of cracks under an external stresses [3], which has an adverse effect on the mechanical properties, and above all on the plasticity and impact strength of the alloy [17].

Mechanical properties of castings can be improved through various processes, such as alloy synthesis (adding alloy additives) [18], modification [19,20] and heat treatment [21]. Precipitation hardening, involving the sequence of solution treatment, quenching and natural (T4) or artificial ageing (T6), is the basic heat treatment applied to silumins containing Cu and/or Mg [22,23,24].

Typical T6 heat treatment comprises three stages:

- solution heat treatment at temperature close to the eutectic one in order to dissolve certain intermetallic phases rich with Cu (Al_2_Cu) and Mg (Mg_2_Si) and formed during solidification, homogenization of alloying elements in a solid solution, and the change in morphology of eutectic silicon [25,26],
- rapid cooling, i.e., cooling down to room temperature to obtain a supersaturated solid solution of dissolved atoms and vacancies,
- natural ageing (at room temperature) or artificial ageing (at elevated temperature) [24,27,28] to precipitation from the solution of the finely dispersed and hardened phase.

In primary Al-Si alloys with low copper content, the main ageing products are metastable modifications of the Mg_2_Si phase (β″and β′), while in the Al-Si alloys containing copper, additional precipitations of the metastable phases—Al_2_Cu (θ″ and θ′), Al_2_CuMg (S), and less frequently, a stable phase θ. The precipitation mechanism of intermetallic phases from supersaturated α(Al) solution is the basis for obtaining changes in the mechanical properties. The strengthening of the AlMgSi alloys occurs due to the release of metastable transition phases and formation of stable equilibrium phases [29,30,31,32]. The sequence of the ageing treatment based on the formation of the Mg_2_Si phase is as follows:

α → GP → β″ → β′ → β(Mg_2_Si)

where:

α—supersaturated solid solution

GP—the Guinier-Preston regions, β″ and β′—metastable transient phases

β(Mg_2_Si)—stable, equilibrium phase

The process commences with formation of spherical GP zones consisting of enrichment of Mg and Si atoms. Then, GP zones elongate and develop into a coherent acicular-shaped β″ phase. Precipitations of the β″ phase increase with time, becoming partially coherent rods (phase β′) and finally incoherent ones, with their matrix in the form of rods or plates (stable β(Mg_2_Si) phase). A maximum alloy strength (peak of the ageing) is reached directly before precipitation of incoherent β(Mg_2_Si) plates. Apelian [27] had observed that the release of very fine β′(Mg_2_Si) during ageing treatment gave a clear improvement in the mechanical properties, while Shivkumar [33] observed a large quantity of fine β″ phases with 2–5 μm in diameter and 10–20 μm in length at peak ageing point, and the length of the β″ phase increased as the ageing progressed. Precipitations of the β″ phase can have an Mg:Si ratio equal to 1:1 [30]. The Mg:Si ratio grows through the GP → β″ → β′ → β zone sequence, and the composition of these phases results from the effect of concentration of Si excess after cooling [34]. The Si can be precipitated in the α(Al) matrix after slight over-ageing, when it is present in a slight excess after cooling [35]. It is presumed that a high concentration of excessive Si after quenching will result in a β″ phase with low Mg:Si ratio [36], as a fraction of available Si is then consumed by the β″ phase formation, while the low concentration of Si remains in the solid solution, in the matrix. This concentration is too low for the Si precipitates to be formed during the initial ageing. The composition of metastable β precipitations changes with progressing ageing, while Si is released into the matrix and Si precipitates are formed later [37]. The morphology of these precipitates also significantly influences the mechanical properties of the alloy. After the T6 heat treatment, a microstructure is realised that is close to ideal for Al-Si alloys: globular particles of the silicon phase uniformly distributed in the aluminum matrix, and the dispersion is hardened by intermetallic compound particles [3]. The appropriate selection of temperatures and times of solution treatment and artificial ageing of the alloy during heat treatment allows to achieve a wide range of mechanical properties.

The purpose of this study was to evaluate the effects of the parameters (i.e., different temperatures and times of solution treatment and artificial ageing processes) of T6 heat treatment, carried out in accordance with the trivalent test plan, on the mechanical properties (tensile strength *UTS*, unit elongation *E*, hardness *HBS*, impact strength *KC*) and microstructure of the EN AC-AlSi11(Fe) alloy.

## 2. Materials and Methods

The study was performed for the near-eutectic EN AC-AlSi11(Fe) alloy, chemical composition of which was determined by spark OES (Table 1).

The increased Fe content in this alloy results primarily from its target application for components produced in the die casting process [18,38].

The alloy was melted in an electric resistance furnace. Then, test pieces for mechanical property tests were cast in permanent moulds. The permanent moulds had been heated to 250 °C prior to pouring; when pouring, the temperature of alloy in the crucible was 720 °C.

**Figure 1:**
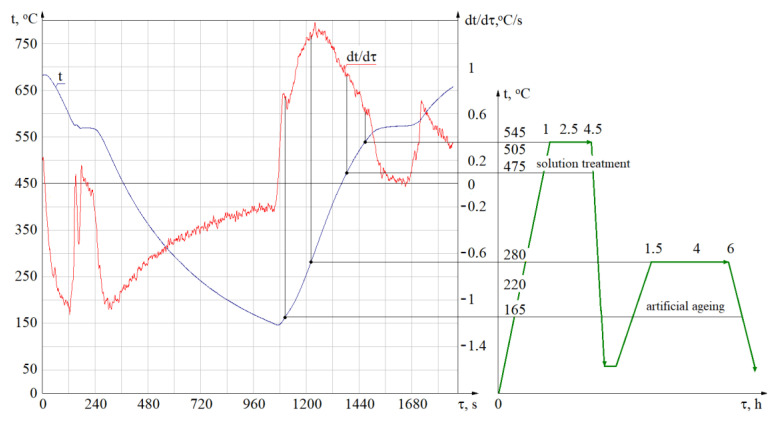
Solution treatment and artificial ageing temperature ranges selected by means of the TDA method.

Solution treatment and artificial ageing temperature ranges were selected after analysing melting and crystallisation curves obtained by means of the thermal derivative analysis (TDA) method (Figure 1).

The times of the individual heat treatment procedures were adopted based on the data provided in the published literature [39,40,41,42,43], bearing in mind the need to limit them for economic reasons.

Due to the number of input variables, the trivalent test plan was adopted (namely three values for each variable parameter), with four variables (solution treatment and artificial ageing temperatures and times) providing 27 combinations in total (Table 2). For each test plan combination, four repetitions were conducted to calculate average values. Measurement uncertainty was also determined in the form of expanded uncertainty (for *k* = 2).

**Figure 2:**
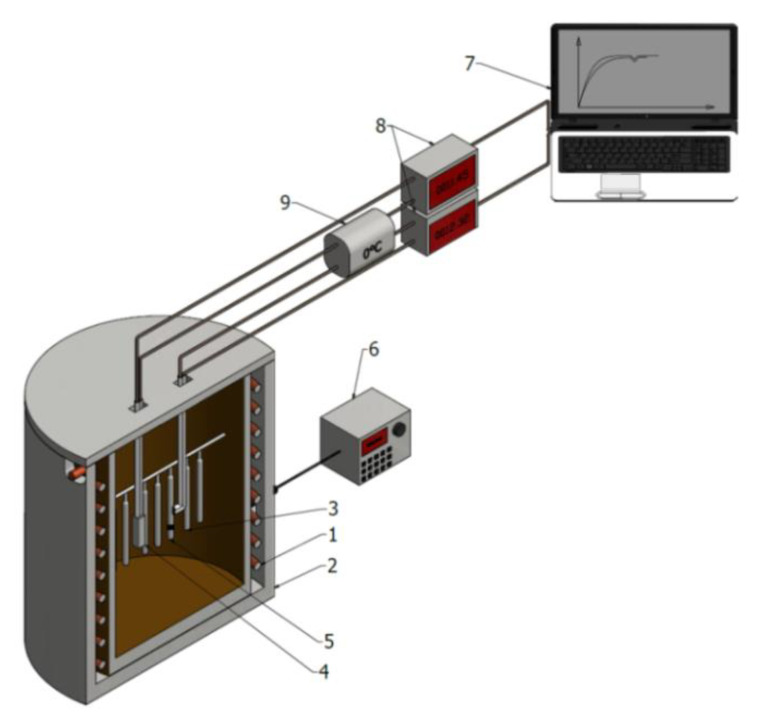
Scheme of the test stand: 1—heating spiral of the furnace; 2—external shield of the furnace; 3—test pieces; 4—thermocouple to measure temperature in the furnace; 5—test piece with the thermocouple; 6—control system of the furnace; 7—computer storing the results; 8—digital micro-voltmeter of V540, V544 type; 9—thermos.

The solution treatment operation was carried out in the test stand, comprising the electric resistance furnace and the measurement and control equipment connected to a computer recording air temperatures in the furnace chamber and control sample temperatures during heating and holding operations (Figure 2).

Temperature measurements were read every 15 s using the type K (NiCr–NiAl) thermocouple. The control sample temperature was maintained within the range of ±5 °C from the set value. After solution treatment, the test pieces were cooled in water at 20 °C. Then, artificial ageing was conducted in a laboratory drier SLN 53 STD, with the temperature measurement accuracy of ±3 °C.

After performing individual heat treatment variants (compliant with the Table 2), the test pieces were prepared for tensile strength *UTS* and unit elongation *E* testing, in accordance with PN–EN ISO 6892–1:2010 [44]. Initially, the diameter of the parallel length of a circular test piece was 10 ± 0.03 mm, and the gauge length was 50 mm [44]. The static tensile tests were performed on the Instron 33R4467 strength testing machine, with 30 kN measuring head, using a 25 mm extensometer gauge (0–2.5 mm, resolution 1 µm, class 1) and the Instron Bluehill 3 software.

The hardness was measured using the Brinell method, in accordance with PN–EN ISO 6506:2008 [45,46]. The measurements were performed using an indenter (Ø10 mm steel ball) under a load of 9807 N sustained for 30 s.

**Figure 3:**
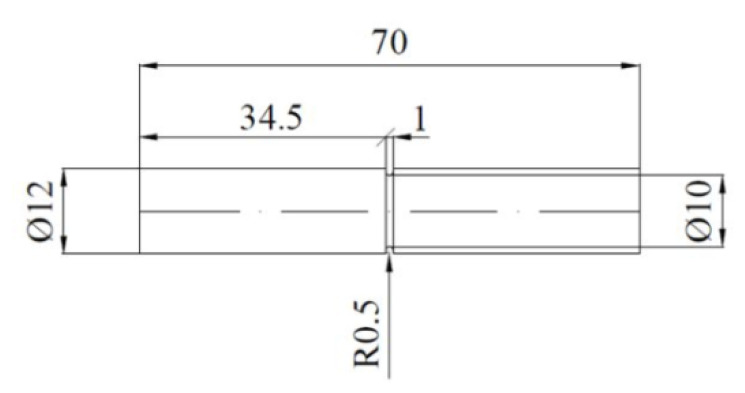
The test piece used for impact strength *KC* testing by a simplified method (dimensions in millimeters) [47].

The impact strength was measured based on the simplified method [47], on the Charpy pendulum machine using notched cylindrical test pieces (Figure 3).

Results obtained after T6 heat treatment of the EN AC–AlSi11(Fe) alloy were implemented into a computer software equipped with the Design of Experiments (DOE) module to determine regression coefficients and equations describing the effect of the heat treatment parameters (*t_p_*, *τ_p_*, *t_s_*, *τ_s_*) on the mechanical properties (*UTS*, *E*, *HBS*, *KC*). The most common approximation method of the regression coefficients is the least squares method. The dominant form of the approximating function formed on the basis of the linear model with respect to the function of base is a second-degree algebraic polynomial, with double products constituting so-called interactions. For the independent variables (*t_p_*, *τ_p_*, *t_s_*, *τ_s_*) as adopted in the test plan (Table 2), it shall have a function that takes the following general form:

(1) $$\begin{array}{c} \hat{y}={\hat{\beta }}_{1}t_{p}+{\hat{\beta }}_{2}\;\tau_{p}+{\hat{\beta }}_{3}\;t_{s}+{\hat{\beta }}_{4}\;\tau_{s}+{\hat{\beta }}_{5}\;t_{p}^{2}+{\hat{\beta }}_{6}\tau_{p}^{2}+{\hat{\beta }}_{7}t_{s}^{2}+{\hat{\beta }}_{8}\;\tau_{s}^{2}+{\hat{\beta }}_{9}t_{p}\tau_{p}\\ +{\hat{\beta }}_{10}t_{p}t_{s}+{\hat{\beta }}_{11}\;t_{p}\tau_{s}+{\hat{\beta }}_{12}\;\tau_{p}t_{s}+{\hat{\beta }}_{13}\;\tau_{p}\tau_{s}+{\hat{\beta }}_{14}\;t_{s}\tau_{s}+{\hat{\beta }}_{15} \end{array}$$

where:

*ŷ*—approximated value of dependent variable (*UTS*, *E*, *HBS* or *KC*)

*t_p_*—solution treatment temperature, °C

*τ_p_*—solution treatment time, h

*t_s_*—artificial ageing temperature, °C

*τ_s_*—artificial ageing time, h

*β_i_*—regression coefficients (*i* = 1, …, 15)

The degree of correlation between the model and the data was determined by the coefficient of determination R^2^ (belongs to <0,1>). When value of the R^2^ is close or equal to 1, it can be said that the practically complete variability of the dependent variable is explained by independent variables of the model. The F test, with the Fisher-Snedecor distribution as its theoretical distribution, was used to assess the effect of independent variables on the dependent variable. Furthermore, the hypothesis testing the significance of each partial regression coefficient was verified. The equations presented further in the study include only the variables with significant contribution in the model. The verification of the model also involved the analysis of residual distribution [48,49].

In addition, for the investigated EN AC-AlSi11(Fe) alloy without the heat treatment, the local chemical composition was determined in the spots under the scanning electron microscope (SEM) performed using energy dispersive x-ray spectroscopy (EDS).

## 3. Results and Discussion

### 3.1. Ultimate Tensile Strength UTS

The tensile strength *UTS* of the raw alloy amounted to 174 MPa. After performing the heat treatment of the alloy, it obtained tensile strength *UTS* ranging from 157 to 265 MPa.

**Figure 4:**
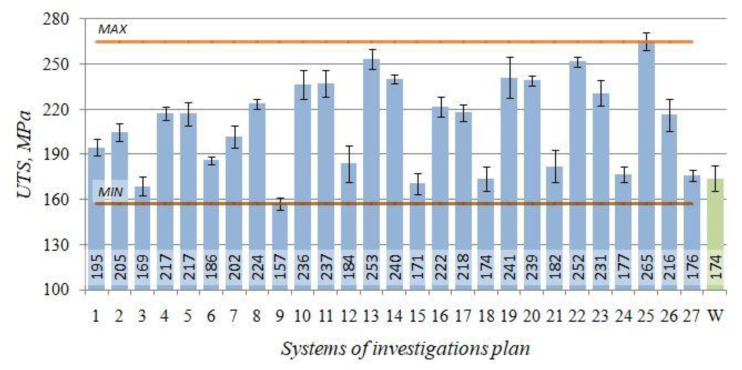
Tensile strength *UTS*—the initial state (W) and after T6 heat treatment (1–27) for the EN AC-AlSi11(Fe) alloy.

The obtained tensile strength *UTS* testing results (Figure 4) for the individual combinations according to the adopted test plan (Table 2) versus the initial state (W) were collated in the form of bar charts.

For the investigated alloy, the highest tensile strength value *UTS* = 265 MPa was obtained after the heat treatment performed according to the test plan combination no. 25 (*t_p_* = 545 °C, *τ_p_* = 4.5 h, *t_s_* = 165 °C, *τ_s_* = 6 h), which indicates over a 50% increase versus the alloy state without the heat treatment (W). Moreover, for the test plan combination no. 13 and 22, the *UTS* values reach over 250 MPa, which indicates over a 43% increase versus the initial state (W) for the shorter time of solution treatment (*τ_p_* = 2.5 h).

For comparison, 232 MPa was obtained by authors of the publication [50], solution treatment of the AlSi11alloy was performed for 6 h at temperatures lower with 25 degrees, and artificial ageing it at 205 °C for 7 h. Instead, Pedersen [51] had received maximal strength amounting to 260 MPa for the AlSi10Mg alloy as early as after 60 min of solution treatment at 540 °C, and 4 h of artificial ageing at 150 °C, while a longer time of the treatment did not lead to increasing the *UTS*. Much lower ultimate tensile strength (215–230 MPa) had been received by authors of the study [52] in case of the 314.0 alloy after extension of the solution treatment time to 8 h, reduced solution treatment temperature (510 °C) and after artificial ageing at a temperature of 155–240 °C for 5 h. Ammar et al. [53] solution treatment the same alloy with addition of 0.4% Mg at temperature 495 °C for 4 h and quenching in hot water (60 °C), and next, naturally ageing, it had received *UTS* similar to the one obtained by Pedersen [51]. The introduction of artificial ageing treatment for 5 h, instead of natural ageing, increased the *UTS* of the alloy to the level of 378 MPa for temperature of 180 °C, and as much as to 401 MPa for temperature 155 °C [54]. In case of the AlSi10Mg alloy, it had been completed many investigations concerning influence of solution treatment and artificial aging on the microstructures and mechanical properties of SLM-produced AlSi10Mg alloy parts, although in case of this technology, the heat treatment did not allow obtaining acceptable mechanical properties [55]. Commonly used in industrial practice alloys with a lower content of silicon, AlSi7Mg and AlSi9Mg are characterised by the tensile strength after the heat treatment at a level similar to the investigated alloy. Pio [56], in case of the AlSi7Mg alloy, after solution treatment at temperature higher with 25 °C for 6 h (artificial ageing at temperature 160 °C for 6 h), obtained *UTS* = 253.5 MPa. The decrease of the artificial ageing temperature to level of 150 °C and shortening of the ageing time to 4 h, as performed by Pedersen [51], resulted in increase of the *UTS* to the level of 270 MPa, whereas the extension of artificial ageing temperature to 230 °C and prolongation of its time to 8 h resulted in a further decrease of the *UTS* to 210 MPa [57]. However, Dobrzański [58], in case of the AlSi9Mg alloy after solution treatment at temperature 525 °C and artificial ageing at 150 °C for 6 h, obtained the ultimate tensile strength amounting to 296 MPa. Increasing ageing temperatures with 15 °C and simultaneously prolonging its time with 10 h had obtained *UTS* at the level of 280 MPa [59]. Even a higher tensile strength (320 MPa) was obtained by Pezda [60] by solution treatment of the alloy at temperature 545 °C for 1.5 h, and ageing it at temperature 180 °C for 8 h.

To illustrate the effect of the heat treatment parameters on tensile strength *UTS*, the regression equation was determined, which takes the form of the relation (2), for which the coefficient of determination of the reduced model is *R*^2^ = 0.92. The statistical significance of the regression equation is τ = 0.05, as *F*_(11;69)_*_calc_* = 78.44 > 1.93 = *F*_(0.05;11;69)_. The distribution of the residuals is close to normal; it can be assumed on this basis that the correlation between the model and the empirical data is very good.

(2) $$\begin{array}{c} {\hat{y}}_{UTS}=8.49372\;t_{p}+46.96824\;\tau_{p}+5.08858\;t_{s}-0.0069\;t_{p}^{2}-2.6251\tau_{p}^{2}\\ -0.00627t_{s}^{2}-0.05412\;t_{p}\tau_{p}-0.00487\;t_{p}t_{s}+0.02548\;t_{p}\tau_{s}\\ -0.02756\;\tau_{p}t_{s}-0.06246\;t_{s}\tau_{s}-2583.44376\;\left[\mathrm{MPa}\right] \end{array}$$

**Figure 5:**
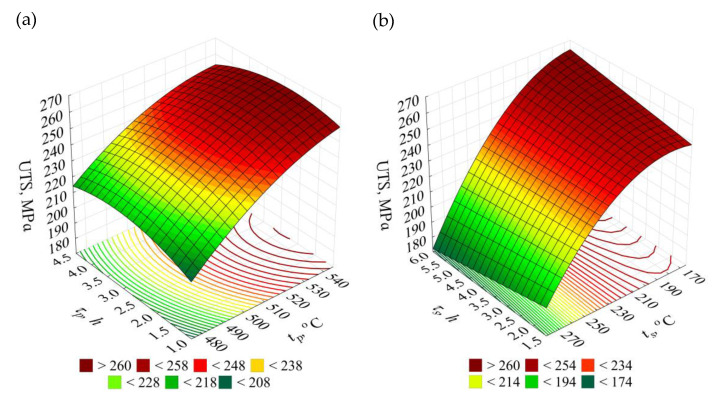
Effects of the T6 heat treatment parameters on tensile strength *UTS* of the EN AC–AlSi11(Fe) alloy for determined (**a**) *t_s_* = 165 °C, *τ_s_* = 6 h, (**b**) *t_p_* = 545 °C, *τ_p_* = 2.5 h.

The relation (2) allowed to present the effect of temperatures and times of solution treatment and artificial ageing on tensile strength *UTS* in the form of 3D and contour graphs (Figure 5).

Parameters of the heat treatment process have very significant influence on change of the *UTS*. Temperature of the solution treatment should be within range of 510–540 °C, while the artificial ageing temperature should be below 200 °C. Increasing the solution treatment temperature caused grain growth and the insoluble phases aggregated and coarsened, resulting in an enhancement of tensile strength [61], while low solution treatment temperature enables obtaining a large amount of homogeneously distributed phases, dispersing and hardening the alloy.

### 3.2. Unit Elongation E

The value of the unit elongation *E*, obtained for the raw alloy amounted to 2.7%, while performed heat treatment that caused the elongation *E* has been changed within a range of 1.5% to 4.2%.

**Figure 6:**
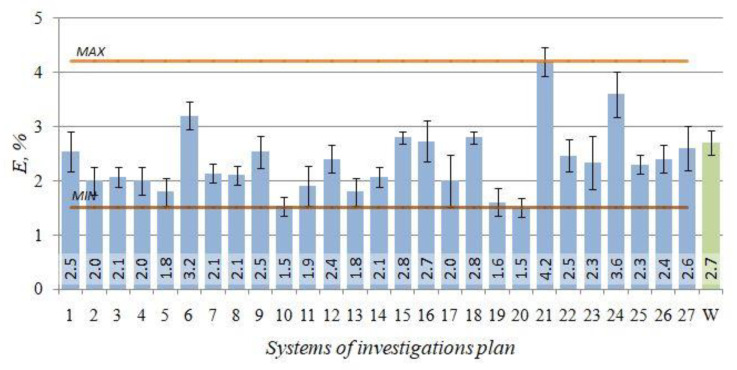
Unit elongation E—the initial state (W) and after T6 heat treatment (1–27) for the EN AC–AlSi11(Fe) alloy.

The unit elongation *E* results obtained for the individual combinations according to the adopted test plan (Table 2) versus the initial state (W) are presented in Figure 6.

Alloy unit elongation *E* reaches the maximum value of 4.2% after the heat treatment performed as per the test plan combination no. 21 (*t_p_* = 545 °C, *τ_p_* *=* 1 h, *t_s_* = 280 °C, *τ_s_* = 6 h) giving an increase by 56% versus the initial state (W). Similar value of the elongation was received by Pedersen [51] for the AlSi10Mg alloy after solution treatment at temperature lower with 5 °C and 4 h artificial ageing at 150 °C. Additionally, 0.4 Mg additive to the 314.0 alloy and its T4 treatment based on solution treatment at temperature 495 °C for 4 h, as well quenching in hot water (60 °C) also enables the obtaining of the elongation at the level of 4.4%. Similar elongation (4.5%) can be also obtained with use of softening annealing, heating the alloy at temperature 370 °C for 8 h [62].

The elongation within limits of 3% after T6 treatment of the AlSi9Mg alloy was obtained by Ananthapadmanaban [59] after solution treatment of a casting for 6 h at temperature 525 °C and artificial ageing at temperature 165 °C for 10 h. It is worth to be noticed, that for the AlSi7Mg alloy, shortening of ageing duration from standard 15 h at temperature 150 °C to 2 h at temperature 170 °C, and solution treatment from 4 h at temperature 535 °C to 2 h at temperature 550 °C enables obtaining 80% of the maximum elongation (7%) [63,64].

The unit elongation *E* lowest value of 1.5% was obtained after T6 heat treatment performed according to the test plan combinations no. 10 (*t_p_* = 505 °C, *τ_p_* = 1 h, *t_s_* = 165 °C, *τ_s_* = 6 h) and no. 20 (*t_p_* = 545 °C, *τ_p_* = 1 h, *t_s_* = 220 °C, *τ_s_* = 1.5 h). This indicates a drop by approximately 45% versus the initial state (W). Even a lower elongation (not exceeding 1%) for the 314.0 alloy with 2% additive of Cu, as well as without this additive, was obtained by Abdelaziz et al. [52] after solution treatment at similar temperature (510 °C) lasting many times longer (8 h), however, and artificial ageing at temperatures 155–240 °C for 5 h. Reduction of solution treatment temperature to 495 °C and solution treatment time in half (to 4 h) with identical parameters of the artificial ageing treatment do not have any effect on improvement of the elongation, which is included within limits of 1.3 to 1.9 % for the alloy without Cu additive, and below 1% in case of the alloy with 2% Cu additive [54].

From the elongation point of view, artificial ageing at low temperature (150 °C) is also disadvantageous for the AlSi9Mg alloy, reducing it from 8.1% directly after the solution treatment for 6 h at 520 °C to 1.2 % for 3 h of the artificial ageing, and 2.3% after 15 h of the artificial ageing [58].

The relation describing the effect of temperatures and times of solution treatment and artificial ageing processes on the unit elongation *E* value is expressed by the Equation (3), for which the determination coefficient of the reduced model is *R*^2^ = 0.78. The value *F*_(12;68)__calc_ = 20.19 > 1.9 = *F*_(0.05;12;68)_.

The equation significance is τ = 0.05. The distribution of the residual values is close to normal. It can be assumed on this basis that the correlation between the model and the empirical data is good.

(3) $$\begin{array}{c} {\hat{y}}_{A_{5}}=-0.178794\;t_{p}-0.125144t_{s}-2.564192\;\tau_{s}+0.000137\;t_{p}^{2}-0.051481\;\tau_{p}^{2}\\ +0.000145\;t_{s}^{2}+0.034897\;\tau_{s}^{2}+0.001153\;t_{p}\tau_{p}+0.000121\;t_{p}t_{s}+0.003555\;t_{p}\tau_{s}\\ -0.001103\;\tau_{p}t_{s}+0.002241\;t_{s}\tau_{s}+64.342632\;\left[\%\right] \end{array}$$

**Figure 7:**
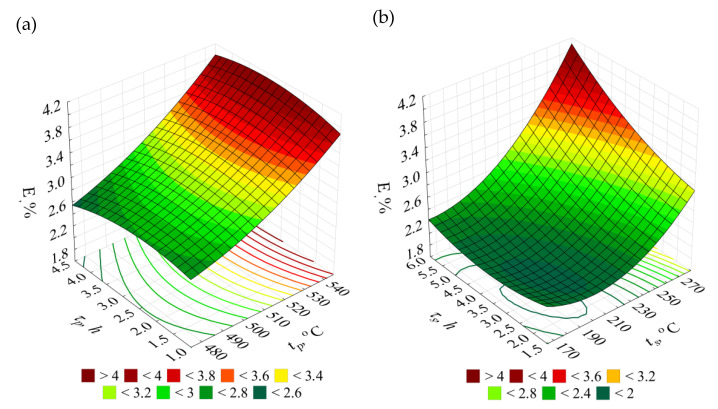
Effects of the T6 heat treatment parameters on unit elongation *E* of the EN AC–AlSi11(Fe) alloy for determined (**a**) *t_s_* = 280 °C, *τ_s_* = 6 h, (**b**) *t_p_* = 545 °C, *τ_p_* = 2.5 h.

The Equation (3) provided the basis for plotting the response surface graphs (Figure 7), illustrating the effect of T6 heat treatment parameters on the unit elongation E.

Relatively high temperature of the solution treatment (slightly below the eutectic temperature) allows spheroidisation of silicon precipitations and dissolution of strengthening components in the matrix of the alloy, combined with long-lasting artificial ageing at temperature 280 °C results in the increase of elongation of the alloy, with simultaneous decrease of its strength, which is mainly due to over-ageing of the alloy, i.e., loss of coherence of the phase precipitated with the matrix, and coagulation of precipitated particles of eutectic silicone.

### 3.3. Hardness HBS 10/1000/30

The *HBS10/1000/30* hardness of the raw alloy amounted to 72 HBS. After performing heat treatment, the obtained hardness of the alloy was within the range of 56 to 104 HBS.

**Figure 8:**
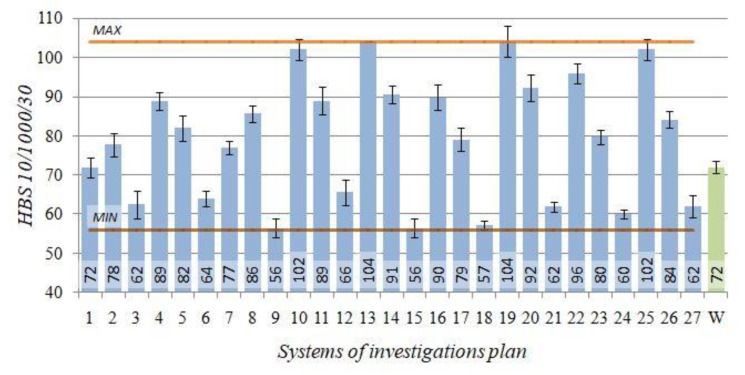
Hardness *HBS 10/1000/30*—the initial state (W) and after T6 heat treatment (1–27) for the EN AC–AlSi11(Fe) alloy.

The obtained results of the EN AC–AlSi11(Fe) alloy hardness *HBS* for the individual combinations according to the adopted test plan (Table 2) versus the initial state (W) are presented in Figure 8.

T6 heat treatment of the EN AC–AlSi11 alloy performed according to the test plan combinations no. 13 (*t_p_* = 505 °C, *τ_p_* = 2.5 h, *t_s_* = 165 °C, *τ_s_* = 4 h) and no. 19 (*t_p_* = 545 °C, *τ_p_* = 1 h, *t_s_* = 165 °C, *τ_s_* = 4 h) resulted in the largest increase in hardness *HBS* (by 44%) versus the initial state (W). Increase at similar level (by 42%) was obtained in case of the systems no. 10 and 25, characterised by identical as the systems specified earlier, low ageing temperature at 2 h longer time of the process.

Hardness at a similar level of 100 HB was obtained by authors of the study [65] for an alloy with similar chemical constitution, solution treatment such alloy for 8 h at temperature 535 °C and artificially ageing it at temperature 180 °C for 3 h. Further prolongation of the artificial ageing time resulted in a slight decrease of the hardness to the level 80–90 HB. Furthermore, increase of the artificial ageing temperature to 200 °C causes reduction of the hardness comparing to the hardness obtained after artificial ageing at 180 °C within range of solution treatment time from 1 to 24 h [65]. Similarly as it happened in case of the tensile strength, and also in case of the hardness, the elements from the AlSi10Mg alloy and produced using additive technologies decrease their hardness after standard heat treatment of T6 type [66].

In case of hypoeutectic alloy of the AlSi7Mg, time needed to obtaining the hardness within limits of 100 HB after solution treatment (540 °C/75 min) amounts as much as 10 h at 170–190 °C (artificial ageing), while in case of temperature 210 °C, it is only 1 h [67]. When the temperature increases above 210 °C, a decrease of the strength was observed [67]. According to Tash [68], meanwhile, in case of the 356.0 alloy, both modified and not modified, the hardness increases together with artificial ageing temperature up to 180 °C (peak temperature); when such temperature is exceeded, a decrease of the hardness occurs at 200 °C and 220 °C (overaging of the alloy). In case of the AlSi9Mg alloy, the hardness increases to 80 to 100 BHN during age hardening (solution treatment: 525 °C/10 h; artificial ageing: 165 °C/10 h) [59].

The functional relationship indicating the effect of the heat treatment parameters on hardness *HBS 10/1000/30* is described by the Equation (4), for which the coefficient of determination of the reduced model is *R*^2^ = 0.94. The significance of the variables in the model is α = 0.05 (*F*_(12,68)__calc_ = 89.19 > 1.9 = *F*_(0.05;12;68)_). The distribution of the residuals is close to normal. Therefore, it can be assumed that the correlation between the obtained model and the empirical data is very good.

(4) $$\begin{array}{c} {\hat{y}}_{HBS}=4.13581\;t_{p}+23.38913\;\tau_{p}+2.26404\;t_{s}+11.58118\;\tau_{s}-0.00322\;t_{p}^{2}\\ -0.33952\;\tau_{p}^{2}-0.00214\;t_{s}^{2}-0.20903\;\tau_{s}^{2}-0.03927\;t_{p}\tau_{p}-0.00284\;t_{p}t_{s}\\ -0.68340\;\tau_{p}\tau_{s}-0.03853\;t_{s}\tau_{s}-1268.14363\;\left[HBS\;10/1000/30\right] \end{array}$$

**Figure 9:**
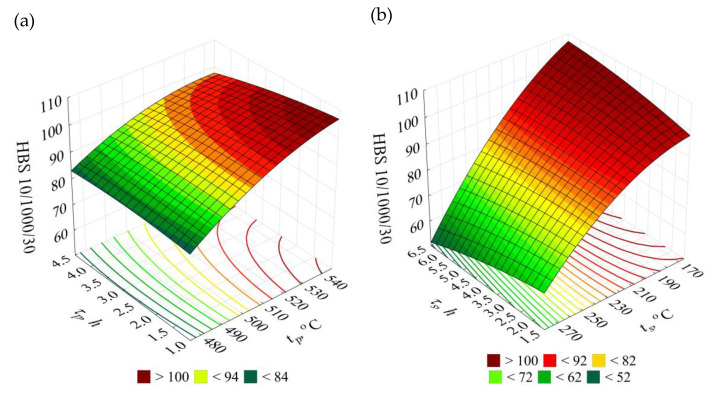
Effects of the T6 heat treatment parameters on hardness *HBS 10/1000/30* of the EN AC-AlSi11(Fe) alloy for determined (**a**) *t_s_* = 165 °C, *τ_s_* = 4 h, (**b**) *t_p_* = 545 °C, *τ_p_* = 2.5 h.

The 3D and contour graphs (Figure 9), obtained on the basis of the Equation (4), show how the hardness *HBS* values evolve depending on the T6 heat treatment parameters, namely temperatures and times of solution treatment and artificial ageing.

In case of the hardness, similarly like in case of the tensile strength, the main role is played by high temperature of the solution treatment (20–30 °C below the eutectic temperature) and low artificial ageing treatment temperature (165 °C) responsible for suitable dispersion degree of precipitations strengthening the alloy. The decrease in the hardness values after the T6 heat treatment can also result from the wide dispersion of eutectic silicon particles [50]. Moreover, according to Iskah et al. [69] area of widely dispersed particles of the silicon is smaller, from its availability for the measuring the intended point of view, comparing to the area of the soft phase α(Al), which results in the lower values of the hardness of the test piece.

### 3.4. Impact Strength KC

The impact strength of the raw alloy amounted to 5.7 J/cm^2^. The impact strength of the alloy after the heat treatment was included within the range from 4.5 to 10.7 J/cm^2^.

**Figure 10:**
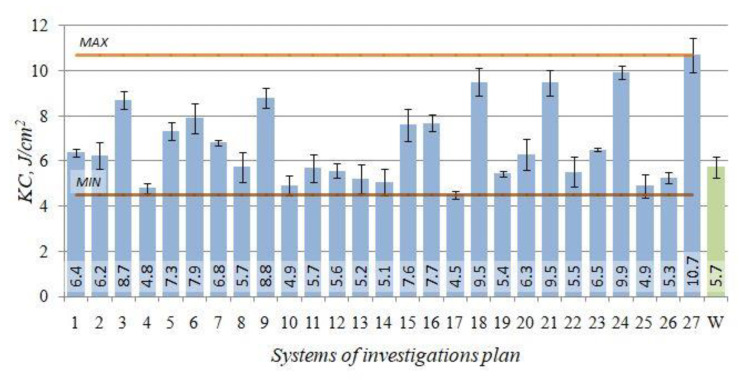
Impact strength *KC*—the initial state (W) and after T6 heat treatment (1–27) for the EN AC–AlSi1(Fe) alloy.

The obtained impact strength *KC* testing results for the test pieces after heat treatment versus the initial state (W) are presented in Figure 10.

For the investigated alloy, the impact strength *KC* maximum value of 10.7 J/cm^2^ was obtained after the heat treatment performed according to the test plan combination no. 27 (*t_p_* = 545 °C, *τ_p_* = 4.5 h, *t_s_* = 280 °C, *τ_s_* = 1.5 h). It is the increase by approximately 88% versus the initial state (W) of impact strength. The increase above 70% relative to the raw alloy was observed for the systems nos. 18, 21, 24 characterised by the longer time of the artificial ageing, and the equally high (as the system no. 27) temperature of the ageing.

In practice, available literature does not deal with topics of an effect of parameters of the T6 heat treatment on impact strength of the investigated alloy. Authors of the publications, except presented results, had also performed studies in range of soft annealing treatment of investigated alloys, which had given increase of the impact strength in case of a raw alloy at a level of 4.5 J/cm^2^ to 6.7–7.7 J/cm^2^ after heating of the material at temperature 370 °C for 5 and 8 h, respectively [70]. Additionally, the studies [71] performed for the AlSi7Mg alloy show that for the solution treatment performed at temperature 545 °C for 1 h and artificial ageing carried out at 280 °C for 5 h, it is possible to generate the impact strength of the order 13 J/cm^2^. Meanwhile, in case of a higher temperatures of the artificial ageing amounting to 325 °C (time: 2 h), the value of the impact strength increases to 27 J/cm^2^ (making solution treatment at 550 °C for 3 h), the lowest values of the impact strength (about 5 J/cm^2^) were obtained for low temperatures and long lasting time of artificial ageing (165 °C for 8 h) [71]. In case of the AlSi9Mg alloy, solution treatment at temperatures 530–550 °C for 1.5 to 3 h and artificial ageing at temperatures above 280 °C for 10–12 h allows obtaining of the impact strength at the level 23–25 J/cm^2^. Instead, a decrease of the artificial ageing temperature to the level of 160 °C drastically reduces its impact strength [60].

The effect of temperatures and times of individual heat treatment procedures on the impact strength *KC* is defined by Equation (5), for which the coefficient of determination of the reduced model is *R*^2^ = 0.83. The statistical significance of the regression equation is *α* = 0.05 (*F*_(9;71)__calc_ = 37.54 > 2.1 = *F*_(0.05;9;71)_). The distribution of the residuals is close to normal. It can be assumed on this basis that the correlation between the model and the empirical data is good.

(5) y^KC=−0.75609 tp−0.292353 ts+0.000694 tp2+0.000396 ts2−0.097927 τs2+0.000229 tpts+0.003009 τpts−0.132103 τpτs+0.004554 tsτs+227.468173 [Jcm2]

**Figure 11:**
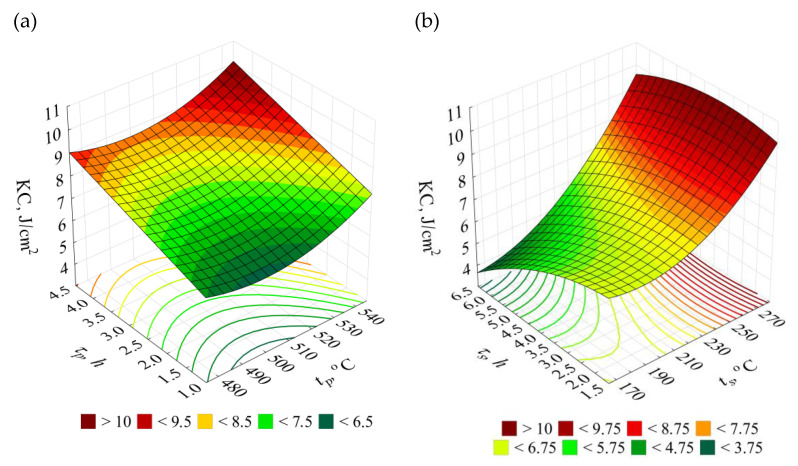
Effects of the T6 heat treatment parameters on impact strength KC of the EN AC–AlSi11(Fe) alloy for determined (**a**) *t_s_* = 280 °C, *τ_s_* = 5 h, (**b**) *t_p_* = 545 °C, *τ_p_* = 2.5 h.

The relation (5) allowed to illustrate the effect of T6 heat treatment parameters on the alloy impact strength *KC* in the form of 3D and contour graphs (Figure 11).

### 3.5. Energy Dispersive X-ray Spectroscopy—EDS

**Figure 12:**
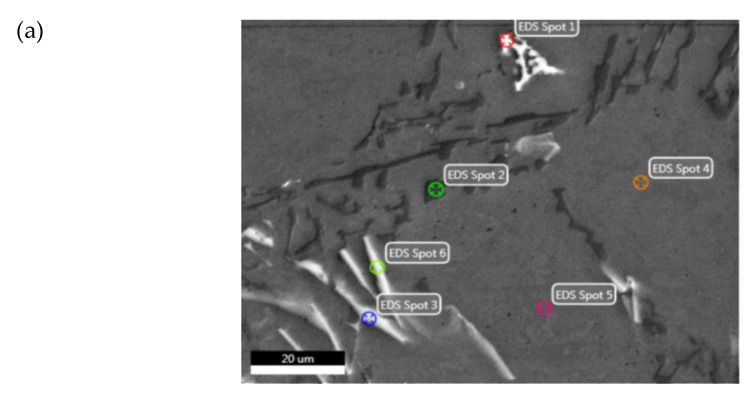

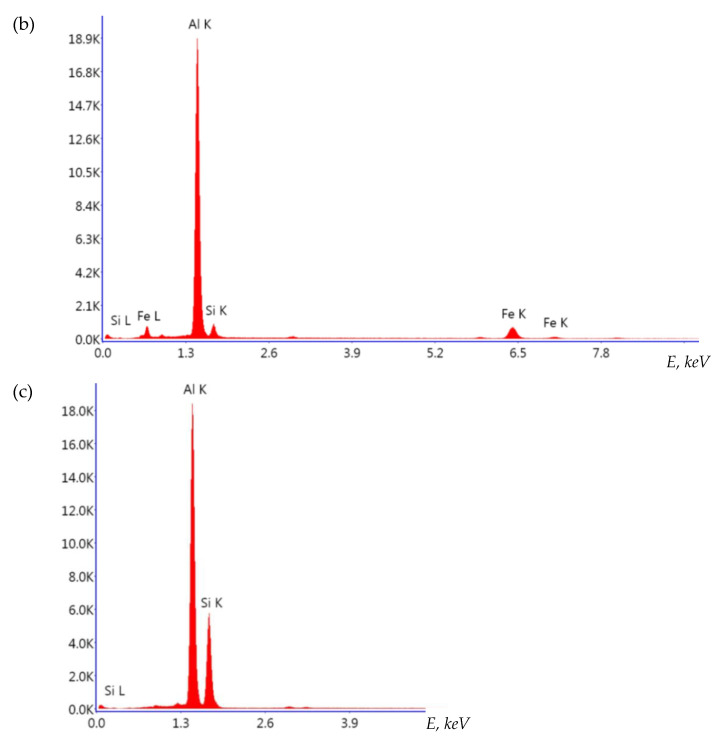
(**a**) EN AC–AlSi11 alloy microstructure in the initial state with marked EDS-analysed microareas. Images of the (**b**) EDS Spot 1, (**c**) EDS spot 2 microareas EDS spectrum.

For the selected microarea of the initial alloy (Figure 12a), the local chemical composition was examined in the spot by the energy dispersive x-ray spectroscopy (EDS) method. Microanalysis results were shown in the form of EDS spectrum. An example sample EDS spectrum (EDS Spot 1 and EDS Spot 2) image is shown in Figure 12b,c.

The analysis of the selected EN AC-AlSi11 alloy microarea microstructure (Figure 12a), EDS spectra and the chemical composition (Table 3) allowed to identify the phase components marked as EDS Spot 1—EDS Spot 6. Table 3 lists the quantitative chemical composition of the EDS-analysed microareas.

The spots marked as EDS Spot 4 and EDS Spot 5 indicate the α(Al) phase. The EDS Spot 2 is the α(Al) + β(Si) eutectic precipitation. EDS analysis also showed the presence of Fe-rich phases in the microarea structure—EDS Spot 1, EDS Spot 3 and EDS Spot 6. The spots marked as EDS Spot 3 and EDS Spot 6 indicate precipitations of the intermetallic β(AlFeSi) phase, which occurs in the form of long platelet-shaped, acicular precipitations visible in the alloy microstructure (Figure 12a). β(AlFeSi) phase has an adverse effect on alloy castability [72], causing a drop in tensile strength, plasticity and impact strength [5,6,73], and it conduces to the initiation of cracks [10] and porosity [74], as well as worsening machinability [75]. The EDS Spot 1 is also an Fe-rich intermetallic phase. This precipitation morphology, visible in the microstructure in the shape of the so-called Chinese script (Figure 12a), enables identifying it as the α(AlFeSi) phase, which occurs during eutectic solidification [76]. Due to its dense morphology, the α(AlFeSi) phase is not as harmful to the alloy mechanical properties as the β(Al5FeSi) phase [77,78,79,80,81,82].

### 3.6. Metallographic Analysis

**Figure 13:**
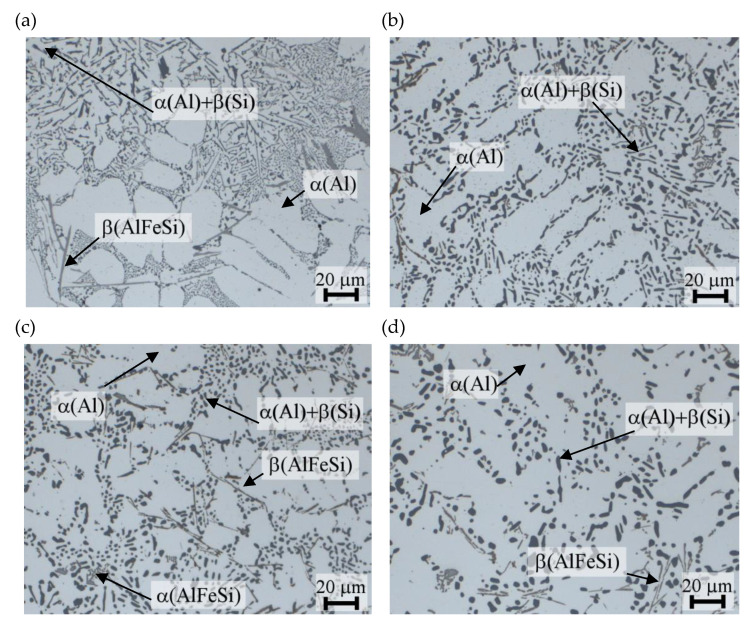
EN AC-AlSi11(Fe) alloy microstructure in the (**a**) initial state and after T6 heat treatment performed according to the test plan combination nos. (**b**) 9 (*t_p_* = 475 °C, *τ_p_* = 4.5 h, *t_s_* = 280 °C, *τ_s_* = 6 h), (**c**) 13 (*t_p_* = 505 °C, *τ_p_* = 2.5 h, *t_s_* = 165 °C, *τ_s_* = 4 h), (**d**) 25 (*t_p_* = 545 °C, *τ_p_* = 4.5 h, *t_s_* = 165 °C, *τ_s_* = 6 h).

The metallographic study was performed to evaluate the microstructure of the investigated EN AC-AlSi11(Fe) alloy. The alloy microstructure in the initial state is shown in Figure 13a. Figure 13b shows the microstructure after the heat treatment performed according to test plan combination no. 9, for which the investigated alloy obtained the lowest values of tensile strength *UTS* = 157 MPa and hardness: 56 HBS, good impact strength *KC* = 8.8 J/cm^2^ and unit elongation *E* = 2.5%. The microstructure after the heat treatment performed according to test plan combination no. 13 is shown in Figure 13c. For this combination, tensile strength *UTS* = 253 MPa, hardness: 104 HBS and unit elongation *E* = 1.8% and impact strength *KC* = 5.2 J/cm^2^ were obtained. The microstructure after T6 heat treatment performed according to test plan combination no. 25 is shown in Figure 13d. For these heat treatment parameters, the highest tensile strength *UTS* = 265 MPa, hardness level: 102 HBS, unit elongation *E* of 2.3% and impact strength *KC* of 4.9 J/cm^2^ were obtained.

The EN AC-AlSi11(Fe) alloy microstructure in the initial state (Figure 13a) is characterized by the presence of eutectic crystals β(Si) (in the form of irregular platelets with sharp edges), which are randomly deposited in the interdendridic spaces of the α(Al) solid solution. The form of the α(Al) + β(Si) eutectic can be defined as acicular. Thin, acicular precipitations of the β(AlFeSi) phase, which have an adverse effect on the mechanical properties of the alloy (*UTS* = 174 MPa, *E* = 2.7%, 72 HBS, *KC* = 5.7 J/cm^2^), are also visible. For the investigated alloy, quantity of the manganese equal to 50% of the iron content was not obtained, which was generally required from transformation of β(Al_5_FeSi) to α(Al_15_(Fe,Mn)_3_Si_2_) point of view [83]. Heat treatment of the alloy causes a change in its microstructure versus the initial state (Figure 13a). Finely divided dendrites of the α(Al) phase are visible in Figure 13b–d. Eutectic silicon precipitations also underwent visible spheroidization and coagulation. This is due to fragmentation happening first at the joints of the branches or the necks of the β(Si) crystals, and then spheroidisation occuring to the fragmented parts, resulting in the formation of elongated or spherical particles [84]. Processes of fragmentation, coagulation and spheroidization, similar to the dissolution, are directly connected with diffusion movements of atoms. Prior solution treatment in the process of the heat treatment, single crystals of β(Si) are split to many relatively small crystals, while each of them endeavours to obtain a spherical shape. This allows obtaining the highest level of mechanical properties. Degree of obtaining spherical shape increases with the increase of the heat treatment duration, and simultaneously the process of coagulation is observed, i.e., the thickening of silicon particles. The thermodynamic driving force, which causes changes in morphology of particles, is the tendency to minimize the total Gibbs free energy of the system [3]. Isolated Fe-rich intermetallic phase precipitations are visible against the α(Al) phase background. Thin and acicular precipitations are the β(AlFeSi) phase [78,79,83,85], and the Chinese script precipitations are the α(AlFeSi) phase [9,86,87]. The minor solubility of iron in α(Al) and acicular morphology of the β(AlFeSi) phase cause that, as a rule, they retain their morphology even after long-lasting heating at temperatures close to the solidus equilibrium, causing the deterioration of the strength and plasticity of Al-Si alloy [88,89] castings and initiation of cracks under load [83].

## 4. Conclusions

The purpose of the study was to identify the effects of T6 heat treatment (precipitation hardening) of the EN AC-AlSi11(Fe) alloy by means of determining its mechanical properties (*UTS, E, HBS, KC*) and to perform a microstructural analysis in the initial state and after T6 heat treatment, according to the trivalent test plan (Table 2) for four variables (temperatures and times of solution treatment and artificial ageing).

On the basis of obtained results, it can be concluded that: the performed heat treatment had a positive effect on a change in the microstructure of the investigated alloy, causing partial spheroidisation and coagulation of eutectic silicon precipitations in comparison with the microstructure of the alloy without the heat treatment. Appropriate selection of the solution treatment and artificial ageing parameters allows for obtaining a several dozen percent increase in the tensile strength, hardness, impact strength and elongation of the AlSi11(Fe) alloy compared to the raw alloy. It is also possible to restrict (to shorten) time of individual operations, which should result in the improved economy of the process. On this basis, it can be concluded that:

- tensile strength *UTS* reaches a maximum value (265 MPa) after the heat treatment performed for *t_p_* = 545 °C, *τ_p_* = 4.5 h, *t_s_* = 165 °C, *τ_s_* = 6 h, which gives an increase by 52% versus the initial value (174 MPa),
- unit elongation *E* reaches a maximum value (4.2%) after the heat treatment performed for: *t_p_* = 545 °C, *τ_p_* = 1 h, *t_s_* = 280 °C, *τ_s_* = 6 h, which gives an increase by 56% versus the initial value (2.7%),
- hardness *HBS 10/1000/30* reaches a maximum value (104 HBS) after the heat treatment performed for: *t_p_* = 505 °C, *τ_p_* = 2.5 h, *t_s_* = 165 °C, *τ_s_* = 4 h and *t_p_* = 545 °C, *τ_p_* = 1 h, *t_s_* = 165 °C, *τ_s_* = 4 h, which gives an increase by 44% versus the initial value (72 HBS),
- impact strength *KC* reaches a maximum value (10.7 J/cm^2^) after the heat treatment performed for *t_p_* = 545 °C, *τ_p_* = 4.5 h, *t_s_* = 280 °C, *τ_s_* = 1.5 h, which gives an increase by 88% versus the initial value (5.7 J/cm^2^).

The obtained response surface models illustrate a tendency to change the mechanical properties (*UTS, E, HBS, KC*) of the investigated alloy in the adopted area of the investigations. Depending on change of temperature and time of the solution treatment and artificial ageing can be used for their preliminary prediction, as well as for obtaining required mechanical properties of the alloy resulting from its heat treatment, which is based on the suitable association of parameters of its solution treatment and the artificial ageing process.

## Figures and Tables

**Table 1:** Chemical composition of the EN AC–AlSi11(Fe) alloy, weight percentage.

Si	Fe	Cu	Mn	Mg	Cr	Ni	Zn	Ti	Al
10.0	0.81	0.37	0.09	0.21	0.01	0.01	0.15	0.09	balance

**Table 2:** The test piece heat treatment plan.

Combination No.	Solution Treatment		Artificial Ageing	
	Temperature (*t_p_*), °C	Time (*τ_p_*), h	Temperature (*t_s_*), °C	Time (*τ_s_*), h
1	475	1	165	1.5
2			220	6
3			280	4
4		2.5	165	6
5			220	4
6			280	1.5
7		4.5	165	4
8			220	1.5
9			280	6
10	505	1	165	6
11			220	4
12			280	1.5
13		2.5	165	4
14			220	1.5
15			280	6
16		4.5	165	1.5
17			220	6
18			280	4
19	545	1	165	4
20			220	1.5
21			280	6
22		2.5	165	1.5
23			220	6
24			280	4
25		4.5	165	6
26			220	4
27			280	1.5

**Table 3:** EDS-determined quantitative chemical compositions of micro-areas, weight and atomic percentage.

Micro-Area	Al, wt. %/at. %	Si, wt. %/at. %	Fe, wt. %/at. %	Phase
EDS Spot 1	74.25/81.75	8.65/9.15	17.11/9.10	α(AlFeSi)
EDS Spot 2	59.25/60.22	40.75/39.78	-	α(Al)+β(Si)
EDS Spot 3	64.24/70.71	19.54/20.67	16.21/8.62	β(AlFeSi)
EDS Spot 4	100	-	-	α(Al)
EDS Spot 5	100	-	-	α(Al)
EDS Spot 6	65.42/71.73	19.02/20.03	15.56/8.24	β(AlFeSi)

## Data Availability

The raw/processed data required to reproduce these findings cannot be shared at this time as the data also forms part of an ongoing study.
